# Novel Therapies for the Treatment of Cardiac Fibrosis Following Myocardial Infarction

**DOI:** 10.3390/biomedicines10092178

**Published:** 2022-09-02

**Authors:** Kamila Raziyeva, Yevgeniy Kim, Zharylkasyn Zharkinbekov, Kamila Temirkhanova, Arman Saparov

**Affiliations:** Department of Medicine, School of Medicine, Nazarbayev University, Nur-Sultan 010000, Kazakhstan

**Keywords:** cardiac fibrosis, scar tissue, anti-fibrotic therapies, cardiovascular diseases, myofibroblasts

## Abstract

Cardiac fibrosis is a common pathological consequence of most myocardial diseases. It is associated with the excessive accumulation of extracellular matrix proteins as well as fibroblast differentiation into myofibroblasts in the cardiac interstitium. This structural remodeling often results in myocardial dysfunctions such as arrhythmias and impaired systolic function in patients with heart conditions, ultimately leading to heart failure and death. An understanding of the precise mechanisms of cardiac fibrosis is still limited due to the numerous signaling pathways, cells, and mediators involved in the process. This review article will focus on the pathophysiological processes associated with the development of cardiac fibrosis. In addition, it will summarize the novel strategies for anti-fibrotic therapies such as epigenetic modifications, miRNAs, and CRISPR technologies as well as various medications in cellular and animal models.

## 1. Introduction

Cardiovascular diseases (CVDs) include all heart and vessel related disorders such as coronary heart disease, cerebrovascular disease, rheumatic heart disease, peripheral artery diseases, and other conditions. Currently, CVDs are among the major causes of death worldwide [1]. According to the World Health Organization, approximately eighteen million people die from cardiac disorders every year, which is equivalent to 39% and 45% of all deaths in the male and female populations, respectively [2]. Recent data from Public Health England shows that high-income countries spend GBP 7.4 billion yearly on CVD treatment. Overall, the forecast for the impact of CVDs on the economy worldwide is disappointing. In the U.S. alone, costs for CVDs are predicted to rise from USD 555 billion in 2015 to USD 1.1 trillion in 2035 [2]. Therefore, developing new advanced approaches and therapies to treat CVDs is of great demand. Importantly, the majority of CVDs result in myocardial fibrosis, which in turn impairs heart function and drastically aggravates cardiac morbidity and mortality.

The term “fibrosis” describes the pathological reparative process occurring in all tissues in the body. It is characterized by the excessive deposition and impaired degradation of extracellular matrix (ECM) proteins, leading to tissue thickness and abnormal tissue remodeling [3]. The main outcomes of fibrosis are rapid wound healing and the reduction in adverse immune responses following injury and infection as well as re-epithelialization and the protection of injured tissue from contaminants. However, fast repair results in the loss of proper anatomy and thus proper functions of the tissue [4]. The formed scar area in the heart is not functional, as cells that replace cardiomyocytes lack a contractile function.

The leading cause of cardiac fibrosis is myocardial infarction (MI) because an adult mammalian heart has limited regenerative capacity following a myocardial injury [5,6]. During MI, dying cardiomyocytes are substituted with a collagen scar, resulting in impaired contractile and mechano-electric functions [7]. Apart from MI, several other pathological cardiac dysfunctions also induce excessive collagen deposition [8]. For example, progressive fibrosis is observed in elderly people and is associated with the development of diastolic heart failure. Myocardial fibrosis can also be formed as a result of persistent pressure load caused by hypertensive heart disease. In addition, hypertrophic cardiomyopathy, idiopathic dilated cardiomyopathy, and metabolic failures are also associated with fibrosis in human and animal models [9,10,11]. Nevertheless, regardless of the nature of the injury, any damage to the heart causes a fibrotic response, which results in cardiac tissue remodeling and dysfunction.

The process of scar formation is orchestrated by a number of cells such as cardiomyocytes, cardiac fibroblasts, endothelial cells, and immune cells [12,13,14]. These cells and their secreted products activate a series of pathways and induce the secretion of paracrine factors, resulting in collagen deposition and myofibroblast transdifferentiation, the two major pathological hallmarks of cardiac fibrosis [15]. Overall, scar formation at the place of necrotic cardiomyocytes leads to myocardial dysfunction and arrhythmia, which increases the risk of tissue rupture and the death of patients with various cardiac conditions [16]. At this time, the precise mechanisms of cardiac fibrosis are still not fully deciphered. The process is complex and requires additional knowledge of the exact mechanisms of cardiac fibroblast activation and their interaction with other cells to understand whether there is a possibility of disabling these cells or modifying their functions during fibrosis [12,17]. Another challenge is the limited potential of the heart to self-regenerate. For example, as much as 25% of cardiomyocytes in the left ventricle die due to acute myocardial injury [18,19]. The inability of cardiomyocytes to proliferate in an adult heart makes it impossible to use therapies directed to totally block scar formation, as there will be no cardiac cells to replenish the lost muscle mass. In addition, the environment after heart injury is highly reactive with a large number of dying cardiac cells and infiltrating immune cells that also make the use of anti-fibrotic drugs challenging.

Overall, the consequences of fibrotic tissue formation on myocardial functions are severe, therefore, understanding the mechanisms of cardiac fibrosis and discovering new potential targets for its therapy may introduce new perspectives for the clinical treatment of heart failure. This manuscript will review the molecular mechanism of scar formation and will focus on novel therapies including epigenetic, clustered regularly interspaced short palindromic repeats (CRISPR), microRNAs (miRNAs), and recent medications directed to treat cardiac fibrosis.

## 2. Cardiac Fibrosis

Cardiac fibrosis is a condition acquired as a result of various heart diseases, including MI, myocarditis, hypertrophy, hypertension, and dilated cardiomyopathy [20,21]. Cellular mechanisms and molecular pathways are common for cardiac fibrosis caused by various disorders. However, their contributions are relatively different due to the specific alterations occurring in cells during various injuries. Overall, the development of fibrosis in the heart is similar to other tissues in the body. It is characterized by the overproduction and impaired degradation of ECM proteins and the accumulation of myofibroblasts [22]. This, in turn, leads to a change in normal cardiac morphology and functions as well as mechano-electric coupling, resulting in increased left ventricular stiffness, delayed systole–diastole cycle, and arrhythmias [13].

There are three types of cardiac fibrosis: replacement fibrosis, when fibrotic tissue substitutes dead cardiomyocytes; reactive fibrosis, featured by the diffuse distribution of collagen in the ECM; and infiltrative fibrosis, presented by the induced deposition of insoluble amyloid, iron, or glycosphingolipids in the heart [23,24]. Replacement fibrosis, which is associated with the loss of healthy cardiomyocytes, keeps the anatomical integrity of the ventricles. In contrast, reactive fibrosis is not related to the death of cardiomyocytes and is rather stimulated by chronic forces such as hemodynamic stress and inflammation, resulting in mechanical stiffness of the myocardial tissue [25]. Thus, although reactive fibrosis is a protective mechanism against raised wall stress, it leads to consequences such as cardiac systolic and diastolic dysfunction, arrhythmias and metabolic impairment, and is associated with a pathological state of fibrosis [26]. Cardiac amyloids, which induces the formation of infiltrative fibrosis in the heart, are pathological extracellular proteins with a stable and fibrous structure. Amyloid fibrils are formed in multiple tissues in the body including the liver, eyes, kidney, and heart [27]. Among all of them, the most important amyloids in the myocardium are transthyretin (TTR) and immunoglobulin light chains [28]. Their deposition into cardiac tissue damages the heart functions by reducing myocardial contractile function and electrical conduction [24].

**Figure 1:**
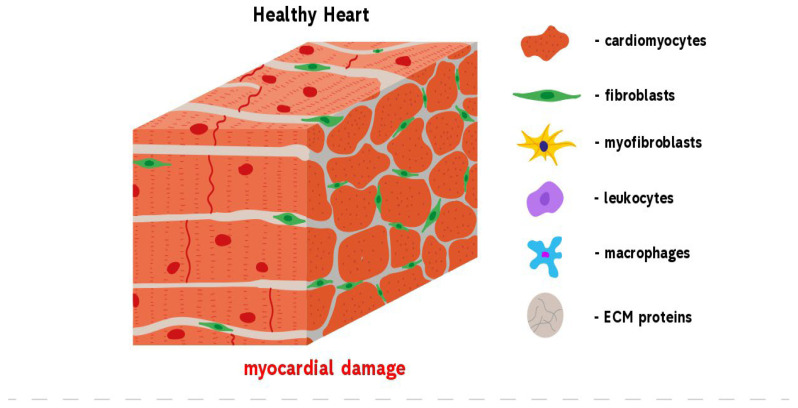

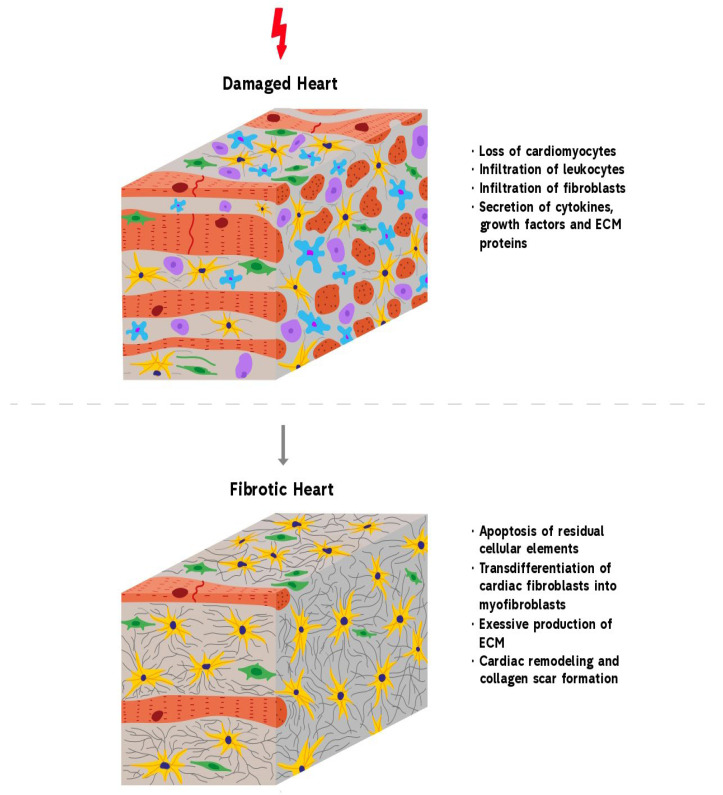
Development of reparative fibrosis in the heart. A healthy heart contains cardiomyocytes that are mainly involved in the contractile function of the heart. An injury to the heart results in myocardial damage. This activates repair mechanisms, leading to the formation of fibrosis. Heart trauma causes the death of cardiomyocytes that express DAMP molecules and by this induce the infiltration of leukocytes and fibroblasts into the damaged area. At the same time, cardiac fibroblasts begin to trans-differentiate into pathological myofibroblasts which, in turn, begin the over-production of ECM proteins. When the wound is cleared from phagocytosing dead cells and matrix debris, resident cells undergo apoptosis and the collagen-based scar is formed.

There are pro- and anti-fibrotic factors that include cytokines, chemokines, hormones, growth factors, and proteases that shift the balance in ECM component production by activating and stimulating myofibroblasts [29]. In turn, secretomes released from myofibroblasts in a paracrine manner stimulate cardiomyocyte hypertrophy, which also leads to decreased capillary density [20]. In particular, massive cardiomyocyte death leads to fibroblast proliferation and differentiation either via direct activation by cell signaling proteins such as miRNAs and matrix metalloproteinases (MMPs) or indirect activation by pro-fibrotic factors produced by endothelial, epithelial, and inflammatory cells [23]. After the death of cardiomyocytes, three overlapping stages, namely, the inflammatory, the proliferative, and the maturation phases, are activated (Figure 1) [30]. Thus, within the first few hours after heart injury, the process of inflammation is initiated: damage-associated molecular pattern (DAMP) signals from dying necrotic cardiomyocytes activate innate immune pathways such as nuclear factor kappa B (NF-kB), leading to the secretion of cytokines and chemokines including IL-1, IL-8, CCL2/MCP-1, and CXCL8 by resident myocardial cells and the infiltration of leukocytes to the damaged area [31]. When the injury is cleared from phagocytosing dead cells and matrix debris, the process is followed by the termination of leukocyte infiltration and their apoptosis in a wound area as well as the secretion of anti-inflammatory molecules such as interleukin-10 (IL-10) and transforming growth factor-β (TGF-β) by macrophages and lymphocytes, which is required for proper transition to the next step and is crucial to avoid left ventricular dilatation [32,33]. The hallmark of the end of the inflammatory phase is the infiltration of the infarct zone with fibroblasts and endothelial cells.

In the next proliferative phase, which usually lasts from a few days to a month, the immune cells such as macrophages, mast cells, and lymphocytes are recruited [34]. They secrete mediators including cytokines, growth factors, and matricellular proteins that initiate transdifferentiation of cardiac fibroblasts into myofibroblasts, whose functions include the change in cell proliferation and migration, the expression of ECM proteins, and the secretion of bioactive molecules [35].

In normal conditions, the heart is composed of endothelial cells, vascular smooth muscle cells, fibroblasts, and cardiomyocytes. The number and proportion of these cells depend on gender, age, and species [20]. For example, a murine heart contains 45% of endothelial cells, 30% of cardiomyocytes, 11% of fibroblasts, 8% of pericytes, and 6% of immune cells [36]. However, an injury can cause the transdifferentiation of cardiac fibroblasts into myofibroblasts. Myofibroblasts have features of both fibroblasts and smooth muscle cells and are normally absent in healthy myocardium [36]. They regulate the production of ECM such as collagen types I and III [37,38]. An excessive production of ECM by myofibroblasts leads to cardiac remodeling [37]. Furthermore, myofibroblasts replace cardiomyocytes that irreversibly die within a few days after the damage, and form scar tissue in the place of injury [17]. Myofibroblast transdifferentiation is a multi-step process, consisting of the development of proto-myofibroblasts, their delivery into the site of lesion, and finally, complete maturation into myofibroblasts [20]. The key feature of mature myofibroblasts is the secretion of α-SMA [37]. The differentiation process is regulated by various cytokines and other signaling molecules such as TGF-β, tumor necrosis factor-α (TNF-α), connective tissue growth factor (CTGF), renin–angiotensin–aldosterone system (RAAS), galectin-3 (Gal-3), endothelin, and IL-11 [23]. Aside from cardiac fibroblasts, other cell types including hematopoietic progenitor cells, pericytes, endothelial, and epithelial cells also show an ability to transdifferentiate into myofibroblasts, however, their role in fibrosis development is unclear [36].

Other cell types including endothelial cells, pericytes, smooth muscle cells, and cardiomyocytes are also involved in the proliferative phase [39]. Activated and proliferated fibroblasts initiate the angiogenic processes by supplying the injured area with oxygen and nutrients and form an extensive vascular network. The proliferation phase is terminated by the release of anti-fibrotic mediators such as IFN-γ, angiotensin AT2-receptor, and CXCL10 to terminate the fibrotic tissue formation [40].

In the final maturation phase, the residual cellular elements including fibroblasts and vascular cells undergo apoptosis in the injured area and the collagen-based scar itself is fully formed (Figure 1). The surrounding tissue is filled with local fibroblasts that constantly respond to morphologically altered infarct zones, where the pressure and volume loads produce activation signals, resulting in the additional production of ECM proteins by local fibroblasts [41]. Over time, myofibroblasts, which secrete ECM proteins to form the scar, transform into matrifibrocytes. Matrifibrocytes secrete cartilage oligomeric matrix proteins and thus support mature scars [42]. Overall, scar formation is a multistep process that involves various cells including infiltrated macrophages, endothelial cells, and lymphocytes as well as transdifferentiated cardiac fibroblasts.

## 3. Molecular Mechanisms of Cardiac Fibrosis

A number of molecular pathways are involved in the pathogenesis of myocardial fibrosis [43]. This, in turn, creates obstacles in the full understanding of the precise mechanisms occurring in this pathological process. Various tools are now implicated in the understanding of the nature of fibrosis including high-throughput genomic and transcriptomic strategies and studies on cell and animal models.

A number of molecular pathways are involved in the pathogenesis of myocardial fibrosis [43]. The TGF-β and WNT signaling pathways, which act as pro-fibrotic mediators in fibrosis formation, are activated in myofibroblasts [20]. Platelet-derived growth factor is also involved in the activation of fibroblasts via the regulation of matrix deposition, pericyte recruitment, and vascular migration [44]. The two critical fibrotic mediators are TGF-β and angiotensin. They stimulate transdifferentiation of cardiac fibroblasts into myofibroblasts and promote the synthesis of collagen in the infarct zone, resulting in repair of the injured site.

### 3.1. The Role of TGF-β in Cardiac Fibrosis

Two main features of cardiac fibrosis include the breakdown of a normal myocardial structure and the overproduction of ECM proteins [45]. The modulation of several growth factor expressions by silencing genes responsible for their production or using neutralizing antibodies can change the progression of fibrotic tissue formation. Thus, the downregulation of TGF-β reduces fibrotic development [46]. This is because TGF-β, together with WNT, which act as pro-fibrotic mediators in cardiac fibrosis formation, are the key regulators of myofibroblast functions [20,47]. TGF-β stimulates the activation of pro-fibrotic genes by increasing Smad2/3 while decreasing the inhibitory Smad 6/7 in myofibroblasts [20]. Smad 2/3, in turn, was reported to be activated in fibroblasts infiltrating the remodeling hearts after injury [48]. TGF-β also increases the deposition of collagens I, III, and VI, and enhances the expression of matrix proteins such as ED-A fibronectin in myofibroblasts through the regulation of plasminogen activator inhibitor, tissue inhibitor of metalloproteinases, and pro-fibrotic cytokine expression [20,49]. In addition, TGF-β suppresses the degradation of ECM proteins by controlling the expression of plasminogen activator inhibitor (PAI)-1 and TIMPs. Moreover, as TGF-β receptors are found in almost all inflammatory cells, TGF-β itself regulates the function of those cells during fibrosis [13].

### 3.2. The Role of RAAS in Cardiac Fibrosis

Following cardiac injury, pro-fibrotic mediators including the components of RAAS activate myofibroblast differentiation, leading to their proliferation and migration as well as the deposition of ECM proteins, which results in the formation of fibrosis [50]. Angiotensin II is generated by macrophages and fibroblasts that migrate into the injured heart area and produce renin and angiotensin-converting enzymes [51]. Once released, angiotensin II stimulates cardiac fibroblast differentiation via either direct activation or through TGF-β1-mediated effects [33]. In particular, angiotensin II causes the generation of reactive oxygen species (ROS), which induce the pro-fibrotic TGF-β1-Smad2/3 signaling pathway activation, collagen type I and III synthesis, and transdifferentiation of cardiac fibroblasts into myofibroblasts. Aldosterone mediates its pro-fibrotic effect via several mechanisms including the stimulation of cytokine and chemokine expression by vascular cells, the induction of macrophage differentiation into fibroblasts, the activation of cardiomyocyte-derived fibrogenic signals, and the stimulation of collagen synthesis [33]. Thus, the inhibition of RAAS facilitates an improvement in the heart functions and prevents further complications of CVDs [52].

Thus, the development of cardiac fibrosis involves numerous molecular processes including hormonal, mechanical, and inflammatory mechanisms such as the activation of RAAS and the expression of fibrogenic growth factors. Their relative significance depends on the nature of fibrosis.

## 4. Anti-Fibrotic Therapies

**Figure 2:**
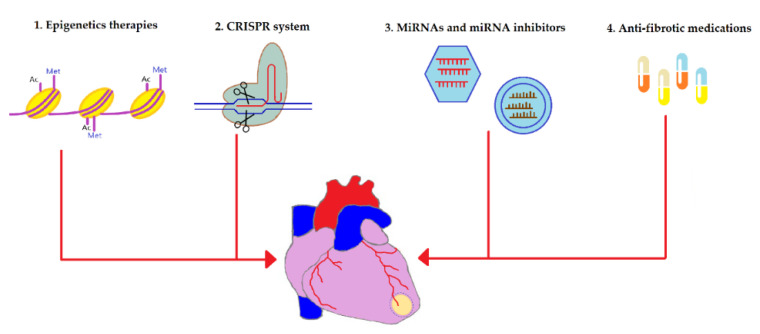
An overview of the novel strategies utilized to alleviate fibrotic remodeling after cardiac diseases. Multiple cardiac pathologies tend to terminate in the replacement of contractile myocardium with the fibrous tissue, one of these diseases is myocardium infarction (infarction area is depicted in yellow). Novel anti-fibrotic therapies target various levels of the pathogenesis of cardiac fibrosis: (1) Epigenetics-based therapies such as histone deacetylase (HDAC) and histone methyltransferases (HMT) have been shown to regulate the gene expression of many fibrosis-associated proteins such as TGF-β1, collagen type I, collagen type III, fibronectin, and many others. Consequently, HDAC inhibitors and HMT inhibitors have been applied to suppress the expressions of those proteins and downregulate fibrosis. (2) Clustered regularly interspaced short palindromic repeats (CRISPR) technology has been used to silence pro-fibrotic genes as well as to introduce “useful” genes into stem cells to enhance their therapeutic potential for myocardial fibrosis. (3) Anti-fibrotic microRNAs (miRNAs) can be utilized to repress the gene expression of fibrosis-mediating genes at the level of mRNA whereas miRNA inhibitors interact with pro-fibrotic miRNA and inhibit them. Viral vectors (shown as hexagons) are generally used to deliver miRNAs while miRNA inhibitors can be transferred using liposomes (depicted as circles). (4) Anti-fibrotic medications such as pirfenidone, angiotensin receptor blockers, and NLRP3 inflammasome inhibitors work by inhibiting enzymes and signaling molecules involved in the pathophysiology of cardiac fibrosis.

Novel anti-fibrotic therapies target various levels of myocardial fibrosis including epigenetic enzymes, genes, translation machinery, and signaling molecules. These therapies are overviewed in Figure 2 and are discussed in detail below.

### 4.1. Epigenetics

Epigenetics-based therapeutic strategies have been successfully utilized to alleviate fibrosis in pre-clinical trials of cardiac, pulmonary, hepatic, and renal diseases [53,54,55]. Epigenetic drugs were shown to reduce fibrosis and improve cardiac function in animal models of MI, ischemia-reperfusion, hypertension, and cardiac hypertrophy [56,57]. However, due to space limitations, we will provide an overview of the use of epigenetic therapies for cardiac fibrosis after MI only. Several groups have demonstrated the importance of epigenetic modifications in the pathogenesis of MI-related fibrosis and proposed novel anti-fibrotic therapies that would target epigenetic regulators [58]. For instance, in a number of studies, targeting histone deacetylases (HDACs) was reported to prevent adverse cardiac remodeling and fibrosis following MI. Wang and colleagues demonstrated that daily intraperitoneal injections of the HDAC inhibitor trichostatin A (TSA) for 8 weeks significantly reduced fibrosis after MI in mice [59]. They also found an improvement in the left ventricular systolic and diastolic functions in the TSA group compared to the controls. It was reported that the cardioprotective effects of TSA were mediated via restoration of autophagosome clearance. Specifically, TSA treatment resulted in a higher expression of LAMP2, an important regulator of autophagosome-lysosome fusion, but decreased levels of autophagic proteins LC3-II, P62, and beclin 1. In contrast, clearance of the autophagosomes was impaired in the control group, which was confirmed by a more than two-fold reduction in LAMP2 expression and 2.5–4.5-fold elevations in the LC3-II, P62, and beclin 1 levels. Importantly, the authors demonstrated that the aforementioned molecular events also happened at the level of the fibroblasts during the cell culture experiments. Thus, TSA repaired autophagosome–lysosome fusion in the neonatal rat ventricular fibroblast primary culture after 4 h of hypoxia treatment.

Another potential target for epigenetics-based therapy of post-MI fibrosis is HDAC4, which was reported by Zhang and colleagues [60]. Using transgenic mice with HDAC4 overexpression, the researchers identified the role of this protein in cardiac development and, most importantly, in MI-induced fibrosis. Compared with the control mice, HDAC4-transgenic mice suffered from substantial interstitial fibrosis, enhanced apoptotic signaling, weakened cardiac function, and reduced capillary density, among other detrimental effects, 3 weeks after MI. Due to its significant role in the pathogenesis of adverse cardiac remodeling following MI, it could be reasonably suggested that the inhibition of HDAC4 might have therapeutic effects on the infarcted myocardium. In particular, the suppression of HDAC4 might potentially decrease fibrosis, prevent apoptosis of cardiomyocytes, increase angiogenesis, and enhance ventricular function after MI overall. In another study, Rhein, a novel class I and II histone deacetylase inhibitor, was demonstrated to block TGF-β1-stimulated fibroblast-to-myofibroblast transition and the transcription of pro-fibrotic genes in primary human ventricular cardiac fibroblasts under sustained hypoxia for four days [61]. Taking into consideration the significant role of TGF-β1 in the pathogenesis of myocardial fibrosis, it can be suggested that the HDAC inhibitor Rhein could become a potential therapeutic approach for MI-induced cardiac fibrosis.

Another group of epigenetic regulators that has been targeted to alleviate post-MI fibrosis is the methyltransferases. In a recent paper, Li and colleagues demonstrated that knockdown of histone methyltransferase DOT1L attenuates fibrosis and improves cardiac function in a murine model of MI [62]. Thus, the degree of post-MI fibrosis was significantly lower in the DOT1L-knockdown group compared to the control group, which was confirmed by the expression levels of collagen type I alpha1 and fibronectin 1 fibrosis markers and Masson’s trichrome staining. Moreover, in the DOT1L-deficient mice, left ventricular function was preserved 4 weeks after MI in contrast to the controls. The authors further reported the important role of DOT1L epigenetic regulation in the progression of fibrosis following MI. First, DOT1L expression was significantly increased in the MI group in comparison to the sham-surgery group. Next, the TGF-β1/Smad3 pathway, which is classically described to be involved in tissue fibrosis, was activated in response to higher levels of DOT1L expression but was suppressed in DOT1L-knockdown cells. Furthermore, it was found that DOT1L methylates lysine-79 of histone H3 (H3K79me2) of the promoter region of the spleen tyrosine kinase (SYK). The latter protein was reported to be elevated in tissue fibrosis. In MI mice, H3K79me2 methylation of the SYK promoter was higher before DOT1L inhibition, whereas DOT1L knockdown reduced this epigenetic modification. Finally, the overexpression of SYK by lentiviral transduction terminated the anti-fibrotic effects of DOT1L inhibition. Thus, it enhanced the expression of fibrosis markers and stimulated the TGF-β1/Smad3 pathway in the fibroblast culture and in DOT1L-deficient MI mice. All of the aforementioned findings indicate the significance of DOT1L epigenetic modifications in the pathogenesis of MI-induced fibrosis as well as provide potential targets for the anti-fibrotic therapies.

Another histone methyltransferase that has been studied as a potential target for anti-fibrotic therapy after MI is G9a. Sung and colleagues reported that suppression of G9a with the UNC0638 inhibitor resulted in a decreased expression of several fibrosis markers, namely, fibronectin, Smad3, and TGF-β as well as reduced fibrotic area, increased angiogenesis, and preserved heart function in a rat model of MI [63]. Interestingly, the amelioration of fibrosis as well as other beneficial effects were synergistically enhanced when G9a suppression was combined with erythropoietin treatment. Erythropoietin itself has been reported to mediate anti-fibrotic effects in myocardial fibrosis. For instance, Liu and colleagues have recently shown that erythropoietin overexpression attenuated cardiac fibrosis in rats with abdominal aortic constriction [64]. The authors revealed that beneficial actions of erythropoietin were generated via the activation of the PI3K/Akt signaling pathway and by lowering Toll-like receptor (TLR) 4 expression, which in turn downregulated the levels of TGF-β1, TNF-α, IL-6, IL-1β, IL-17A, MMP-2, and MMP-9. In summary, certain HDAC and methyltransferases have been identified as potential mediators of fibrotic cardiac remodeling after MI and other CVDs. The obtained data are summarized in Table 1. It was, therefore, reasonably suggested that the inhibition of these epigenetic regulators might present a promising approach to treat fibrosis after MI, which was indeed demonstrated by recent studies. Nevertheless, it should be mentioned that complete inhibition of fibrotic response after MI and other cardiac diseases might have detrimental consequences rather than therapeutic effects due to the fact that fibrosis has important protective effects, namely, it helps maintain the heart’s structural integrity and secures the organ from rupture [39].

### 4.2. CRISPR

CRISPR technology has been demonstrated to attenuate liver, renal, and pulmonary fibrosis in animal models [65,66,67,68]. Although CRISPR has been utilized to model and treat CVD caused by genetic mutations, the use of this approach as a therapy for acquired diseases associated with cardiac fibrosis is quite limited [69,70]. Nevertheless, the latest studies report encouraging results of CRISPR technology for the treatment of fibrotic cardiac remodeling after MI. In a recent study by Park and colleagues [70], a CRISPR/Cas9 system-based on in vivo genome editing was used to treat MI in a murine model. Specifically, CRISPR was designed to mediate inactivation of the miR34a gene, whose deletion has been shown to reduce cardiomyocyte death and myocardial fibrosis. In order to ensure more efficient targeted delivery, the researchers created CRISPR/Cas9-conjugated magnetic nanoparticles that could be directed to the heart using an external magnetic field following intravenous injection. Treatment with CRISPR/Cas9-combined magnetic nanoparticles resulted in decreased fibrosis two weeks after MI, which was demonstrated by Masson trichrome staining and expression levels of fibrosis marker genes, namely, TGF-β2, FN, CTGF, COL1, COL3, and Postn. Moreover, the treatment significantly increased the proliferation of cardiomyocytes and overall improved contractile function of the left ventricle. In another study, Jiang and colleagues used CRISPR to reprogram fibroblasts into cardiovascular progenitor cells, which ameliorated heart function and reduced myocardial fibrosis after transplanting them into mice with MI [71]. In particular, mouse extracardiac fibroblasts were reprogrammed into cardiovascular progenitor cells in vitro by activating *Gata4*, *Nkx2.5*, and *Tbx5* genes using lentiviral transduction with the CRISPR activation system. After injection into the infarction area of the mouse heart, these cardiovascular progenitor cells differentiated into the cells of the cardiac tissue. In particular, 39% of the engrafted cells differentiated into CD31+ endothelial cells, 36% of the cells became cTnT+ cardiomyocytes, and 24% of the cells gave rise to α-SMA+ smooth muscle cells. Importantly, treatment with CRISPR-induced cardiovascular progenitor cells significantly reduced the post-infarction scar area and recovered cardiac function, indicating that this therapeutic strategy has the potential to become one of the novel anti-fibrotic and regenerative therapies for MI.

The CRISPR system has also been used indirectly to optimize cellular therapy for MI. Cho and colleagues utilized CRISPR/Cas9 to integrate the LEF1 gene into human umbilical cord blood-derived mesenchymal stem cells (hUCB-MSCs) in order to enhance their therapeutic efficiency [72]. The LEF1 gene, which is involved in cellular proliferation, survival, and differentiation, was introduced into hUCB-MSCs using transfection with Lipofectamine 3000 and then inserted into an adeno-associated virus integration site 1 with the help of CRISPR/Cas9 tools. As the authors state, CRISPR/Cas9 was chosen over viral gene editing techniques to avoid potential off-target effects and tumorigenesis. It was found that the LEF1 gene was precisely integrated into the targeted site and continuously expressed by the genetically modified cells. Importantly, CRISPR/Cas9-edited hUCB-MSCs showed greater survival after implantation into the infarcted hearts of rat models using the UpCell cardiac patch system. Furthermore, compared with the non-engineered hUCB-MSCs and no treatment groups, therapy with LEF1-knockin cells resulted in improved overall survival, enhanced cardiac function, increased vessel density, and reduced fibrosis after MI. Specifically, infarction of the myocardium and the formation of fibrotic tissue happened 52% less in the LEF1-expressing hUCB-MSCs-treated animals versus only 21% less in the non-modified hUCB-MSCs in comparison to the control group. In another interesting study, CRISPR engineering was employed to improve the survival and the therapeutic potential of bone marrow-derived mesenchymal stem cells (BM-MSCs) for the treatment of MI in the context of diabetes mellitus [73]. To be specific, the researchers used the CRISPR/dCas9 activation system to induce the overexpression of IL-10 in BM-MSCs. Transcription activation of IL-10 was successfully accomplished by CRISPR/dCas9, which was demonstrated by a high and stable expression of this cytokine in vitro as well as after BM-MSC transplantation to a murine model of MI. Moreover, IL-10 overexpressing BM-MSCs mediated regenerative and reparative effects on the infarcted myocardium by reducing scar tissue, bettering post-infarction heart function, inhibiting apoptosis, and enhancing angiogenesis. It is important to point out that one of the findings in this study was the suppression of inflammation by the IL-10-engineered BM-MSCs. Namely, the transplanted cells caused decreased recruitment of the CD68+ immune cells and suppressed the expression of IL-1β, TNF-α, IL-6, and MCP-1 pro-inflammatory cytokines. Since inflammation is believed to be a critical contributor to fibrotic remodeling after MI, it could be suggested that in this study, IL-10-engineered BM-MSCs reduced fibrosis due to their anti-inflammatory actions [74].

Overall, there is some hesitancy in implementing CRISPR technology for the treatment of cardiac fibrosis due to numerous challenges such as the postmitotic nature of cardiomyocytes, difficulties in achieving targeted delivery, restricted cargo capacity of delivery systems, possible off-target mutagenesis, and others [69,75]. Despite the above-mentioned limitations, data from several studies are quite encouraging. In particular, the CRISPR system was utilized to directly edit genes involved in MI and fibrosis as well as to enhance the efficiency of cell mediated therapy for MI, which is summarized in Table 1.

**Table 1:** The epigenetics and clustered regularly interspaced short palindromic repeats (CRISPR) used for the treatment of cardiac fibrosis.

**Strategy**	**Treatment**	**Outcome**	**Reference**
Epigenetics	Histone deacetylase inhibitor trichostatin A	Reduced fibrosis and improved systolic and diastolic functions a murine MI model	[59]
	Class I and II Histone deacetylase inhibitor Rhein	Inhibited TGF-β1-induced fibroblast-to-myofibroblast transition and transcription of pro-fibrotic genes in primary human ventricular cardiac fibroblasts culture under sustained hypoxia	[61]
	Histone methyltransferase DOT1L inhibition	Alleviated fibrosis, reduced expression of collagen type I alpha1 and fibronectin 1 and improved cardiac function in mice with MI	[62]
	Histone methyltransferase G9a inhibition	Decreased expression of several fibrosis markers such as fibronectin, Smad3, and TGF-ß; reduced fibrotic area; increased angiogenesis; and preserved heart function in a rat model of MI	[63]
CRISPR	CRISPR-Cas9-mediated inactivation of miR34a gene	Decreased fibrosis, enhanced proliferation cardiomyocytes, and improved heart function	[70]
	CRISPR-mediated reprogramming of fibroblasts to cardiovascular progenitor cells	Differentiation of reprogrammed fibroblasts to endothelial cells, cardiomyocytes, and smooth muscle cells; reduced scar size; and restored cardiac function in a mouse model of MI	[71]
	CRISPR/Cas9-mediated integration of LEF1 gene into human umbilical cord blood-derived mesenchymal stem cells	Improved survival, enhanced cardiac function, increased vessel density, and decreased fibrosis after MI	[72]
	CRISPR/dCas9 activation system-induced overexpression of IL-10 in bone marrow-derived mesenchymal stem cells	Reduced scar tissue, improved heart function, suppresses cardiomyocyte apoptosis, and enhanced angiogenesis	[73]

### 4.3. miRNAs

MiRNAs are small (about 22 nucleotides), single-stranded, non-coding RNA molecules involved in the post-transcriptional regulation of genes implicated in various biological processes [76]. A considerable amount of evidence points that miRNAs are extensively involved in the regulation of cardiac remodeling after different CVDs [77,78]. Depending on the effects of fibrosis, miRNAs could be classified into pro-fibrotic and anti-fibrotic, and each of the two and the associated pathways that could be targeted to alleviate cardiac remodeling will be reviewed below.

#### 4.3.1. Pro-Fibrotic miRNAs

Pro-fibrotic miRNAs promote the increased expression of ECM components and other fibrosis-related proteins in different tissues, and therefore, could serve as an attractive target for therapeutic modifications in cardiac diseases such as MI, atrial fibrillation, cardiac ischemia, and others [79,80,81,82]. One of the first examples to be considered was miR-27b, which has been shown to stimulate hypertrophy and promote heart failure, whereas its inhibition alleviates cardiac dysfunction [83,84]. Further findings have indicated that a TGF-β1/Smad signaling pathway plays a key role in the regulation of miR-27b, since TGF-β1 interfered with the activity of the miR-27b promoter and reduced the growth of hypertrophic cells, while smad4 knockout, in contrast, stimulated hypertrophy [85]. Later, another study identified a different pathway, FBW7/Snail, regulated by the same miRNA [86]. The results suggest that miR-27b may target the FBW7 ubiquitin ligase and suppress Snail degradation, which promotes cardiac fibroblast proliferation and ECM synthesis, leading to myocardial fibrosis. In particular, miR-27b transfection of cardiac fibroblasts substantially lowered the expression of FBW7 at 2, 6, and 12 days in the peri-infarct area of rats with MI when compared with the sham-operated animals. In addition, the same manipulations were shown to suppress the luciferase activity of FBW7, which, in turn, was restored upon the inhibition of miR-27b by its antagonist, suggesting that miR-27b suppresses FBW7. Furthermore, it was reported that FBW7 expression in the cardiac fibroblasts inhibited Snail induction, collagen I and III, and MMP-9. Experiments performed using antagomir-27b also showed that downregulation of miR-27b has beneficial effects on cardiac remodeling as it leads to a highly significant reduction in fibrotic tissue in rat MI models [86]. Similarly, miR-96 has been reported to have a pro-fibrogenic function in the context of atrial fibrosis [87]. Thus, Su and colleagues demonstrated that its inhibition was able to suppress Ang-II-induced proliferation, migration, and collagen production by mouse cardiac fibroblasts, as evidenced by CCK-8 and Transwell migration assays, reduced inflammatory infiltration, and decreased deposition of collagen I and III. Through analysis of a publicly available database, the authors were able to identify a potential gene target of miR-96, Kruppel-like factor 13 (Klf13), which has previously been shown to be associated with heart development and cardiomyocyte protection against DNA damage and apoptosis. According to the luciferase reporter assay results, HEK293T cells cotransfected with the Wt-3′UTR reporter and miR-96 had significantly lower luciferase activity compared to the negative controls, whereas the activity of the mutant reporter gene was not affected. Additionally, the overexpression of miR-96 has been shown to suppress Klf13 expression, whereas knockdown had the opposite effect. Collectively, these results suggest that Klf13 is a functional target of miR-96 and that the effects of miR-96 on atrial fibrosis are mediated by Klf13 downregulation [87].

Another miRNA involved in fibrogenesis after CVD is miR-99b-3p. Recent work showed that miR-99b-3p was upregulated in a cardiac fibrosis model induced by angiotensin II [88]. Moreover, forced overexpression of this miRNA led to an increase in the expression of pro-fibrotic biomarkers (fibronectin, collagen I, vimentin, and α-SMA) and the proliferation of cardiac fibroblasts and migration, whereas its inhibition had the opposite effect. In addition, target prediction software revealed a negative correlation between miR-99b-3p and GSK-3 beta, serine threonine kinase implicated in the pathogenesis of diabetes, cancer, inflammation, and CVDs. A quantitative real-time polymerase chain reaction analysis showed that the level of GSK-3β mRNA in cardiac fibroblasts was not affected by miR-99b-3p. However, at the protein level, GSK-3β expression was markedly reduced by the miR-99b-3p mimic, which implies its regulatory effect by suppressing translation. Importantly, other studies have demonstrated that GSK-3β exerts its anti-fibrotic effect by interacting with Smad3 and inhibiting its activation [13]. In agreement with this, the administration of the miR-99b-3p mimic led to enhanced levels of phosphorylated smad3. The same effect was observed upon the inactivation of GSK-3β. Overall, it could be proposed that miR-99b-3p can promote cardiac fibrosis by downregulating GSK-3β, and therefore can lead to the activation of the downstream pro-fibrotic effector Smad3 [88].

MiR-1202 is yet another miRNA that has been reported to have pro-fibrotic properties. In a recent work by Xiao and colleagues, it was reported that TGF-β1-stimulated human cardiac fibroblasts had a high expression of miR-1202, which was time and dose dependent [89]. According to their observations, transfection of the fibroblasts with the miR-1202 mimic increased the levels of collagen I and III, α-SMA, and the Smad2/3 protein as well as promoted Smad2/3 phosphorylation, subsequently leading to the deposition of ECM. Next, using the TargetScan software, the 3′-UTR region of the nitric oxide synthase gene was identified as a target site for miR-1202. As a proof-of-concept, the miR-1202 mimic was shown to reduce the luciferase activity of the NOS1 WT 3′-UTR relative to that of the control group. Moreover, the nNOS protein expression in human cardiac fibroblasts was also shown to be inhibited by miR-1202 mimic transfection and TGF-β1 treatment, but this trend was reversed when the miR-1202 inhibitor was used. In summary, due to their pro-fibrotic effects, the aforementioned miRNAs could be considered as attractive targets for anti-fibrotic therapies of various heart diseases.

#### 4.3.2. Anti-Fibrotic miRNAs

Anti-fibrotic miRNAs can also be considered as therapeutic targets for CVDs as their overexpression was reported to suppress fibrosis-associated cardiac remodeling [90,91]. For instance, miR-150 is involved in the suppression of fibrosis after MI. In a recent study by Tian and colleagues, miR-150 significantly inhibited the expression of myocardial fibrosis-related proteins such as col1α1, col1α2, col3, and α-SMA in the border zone of MI [92]. In addition, miR-150 treatment improved survival and suppressed apoptosis of cardiomyocytes. In a related study, the mechanism by which miR-150-5p prevents the progression of myocardial fibrosis was described [93]. First, miR-150-5p was shown to have inhibitory effects on the expression of fibrosis-related proteins such as MMP-13, collagen I and III, and to induce apoptosis of human myocardial fibrosis cells generated by Trypanosoma cruzi infection. In contrast, another known fibrosis regulator, namely, early growth response 1 (EGR1), was found to improve cell proliferation and enhance the expression of three fibrosis-related proteins, MMP-13, collagen I, and collagen III, in the myocardial fibrosis cell culture. Next, the upregulation of miR-150-5p reversed the effect of EGR1, pointing to the possible mechanism through which they directly interacted with each other. At the same time, EGR1 knockdown retarded myocardial fibrosis while silencing miR-150-5p exacerbated fibrotic expansion, as evidenced by H&E and Masson’s trichrome staining. Putting all of this together, it was concluded that the inhibition of EGR1 by miR-150 might become an effective method for cardiac fibrosis therapy [93]. Similarly, miR-1954 has also been determined to play a critical role in cardiac fibrogenesis [94]. Specifically, heart-specific overexpression of miR-1954 was demonstrated to be protective in a mouse model of cardiac hypertrophy and remodeling generated by angiotensin II infusion. Namely, miR-1954 attenuated cardiac remodeling, reduced systolic blood pressure, and decreased the expression of cardiac hypertrophy (NppA, NppB, beta-MHC) and fibrotic marker genes (col1a1, col3a1, and col4) [94]. The investigators further proposed the potential target of miR-1954, namely, thombospondin 1 (THBS1), since cardiac fibroblasts transfected with miR-1954 had lower expression levels of THBS1, whereas the inhibition of miR-1954 had the opposite effect. Thus, it was suggested that the enhancement of miR-1954 levels could be another promising treatment strategy, as it targets the THBS1 gene involved in the promotion of cardiac fibrosis [94]. Yang and colleagues reported a different miRNA type that could negatively regulate cardiac fibrosis [95]. In their study, miR-489 suppressed isoproterenol induced cardiac fibrosis in rats. To be specific, miR-489 downregulated pro-fibrotic markers such as col1a1, α-SMA, and HDAC2 and inhibited the viability and differentiation of cardiac fibroblasts. Importantly, HDAC2 was identified as a direct target of miR-489 by using the computational prediction software, TargetScan. This was further confirmed by a reverse transcription-quantitative polymerase chain reaction analysis, which demonstrated a decrease in the HDAC2 expression in cells transfected with the miR-489 mimic and a corresponding increase in cells transfected with the miR inhibitor. They also determined a reduction in the colA1 and α-SMA levels with HDAC2 silencing in the siHDAC-transfected group compared to siNC, providing additional clues to the mechanism of action of miR-489. Another anti-fibrotic miRNA that was recently investigated in the context of CVDs is miR-30d. In a murine model of MI, overexpression of miR-30d in the heart ameliorated left ventricular function, reduced cardiac fibrosis, and downregulated fibrotic markers such as α-SMA [96]. It is important to mention that miR-30d overexpression was also associated with inhibitory effects on apoptosis. Further investigations revealed that integrin α5 was a direct target of miR-30d, which is involved in the regulation of fibrogenesis. Similarly, miR-145 has also been identified as a suppressor of MI-induced cardiac fibrosis in rat models [97]. Thus, the injection of adenovirus expressing miR-145 in the infarcted zone significantly reduced fibrous tissue formation and collagen synthesis. Moreover, improvement in left ventricular functions was observed upon treatment with miR-145. Regarding the mechanism of action of miR-145, it was found that SOX9, which plays a role in cardiac fibrogenesis, was directly repressed by miR-145. Furthermore, the study showed that SOX9 negatively regulated PTEN and promoted PI3K, AKT, and GSK-3beta signaling and collagen I/III secretion, whereas a contrary effect was observed after miR-145 overexpression, suggesting an inhibitory role of miR-145 on SOX9 and its downstream PI3K/AKT pathway. Overall, this study provided evidence for the involvement of SOX9 and miR-145 in cardiac fibrogenesis, which can be further used to develop new strategies to combat cardiac fibrosis [97]. Summarizing all of the data, some miRNAs have been identified as potential mediators of cardiac fibrosis, while others have been shown to have the opposite effect. Taking this into account, the up- and downregulation of these miRNAs may provide novel strategies for CVDs associated with fibrotic cardiac remodeling. We summarize some of the related studies in Table 2.

### 4.4. Anti-Fibrotic Medications

Differentiation of pathological myofibroblasts from cardiac fibroblasts is the major hallmark of cardiac fibrosis [98]. Thus, controlling this process is crucial for attenuating the pathology of fibrosis. CTGF, TGF-β, RAAS, Gal-3, TNF-α, endothelin, and IL-11 are either directly or indirectly involved in the differentiation of fibroblasts into myofibroblasts. Therefore, the current therapies are mainly focused on targeting these pro-fibrotic agents in order to attenuate the development of cardiac fibrosis in damaged hearts. These therapies include, but are not limited to, drugs focused on the inhibition of RAAS, TGF-β, CTGF, Gal-3, and NLRP3 expression and production.

#### 4.4.1. Renin–Angiotensin–Aldosterone System (RAAS) Inhibitors

RAAS inhibitors are widely used to target cardiac fibrosis. Drugs such as lisinopril, losartan, amlodipine, and spironolactone have proven their anti-fibrotic effect on cardiomyocytes [23,99,100]. A recent study demonstrated that a new first-in-class angiotensin receptor inhibitor, sacubitril/valsartan, can suppress the effect of RAAS during cardiac remodeling by blocking angiotensin II type 1 receptors and activating vasoactive peptides through the inhibition of the neprilysin enzyme, which is responsible for their degradation [101]. Sacubitril/valsartan prevented maladaptive cardiac fibrosis and dysfunction by blocking cardiac fibroblast activation and proliferation in a mouse model of pressure overload–induced hypertrophy [102]. Moreover, the effects on exosome production and content isolated from human induced pluripotent stem cell-derived cardiomyocytes by sacubitril/valsartan demonstrated that it decreases myocardial fibrosis via the downregulation of exosomal miR-181a, resulting in the attenuation of myocardial fibrosis and hypertrophy in a rodent model of chronic MI [103]. Another recently identified RAAS inhibitor, alamandine, demonstrated that it could decrease the density of cardiac fibrosis and reduce the expression of fibrotic proteins (CTGF, collagen I (COL1A1), and MMP-9) in a spontaneous hypertensive rat model [104]. Furthermore, alamandine blocks the increase in ERK1/2 phosphorylation and restores the level of 5′-adenosine monophosphate-activated protein kinase (AMPK) α phosphorylation, preventing cardiac hypertrophy and fibrosis in a mouse model of cardiac remodeling induced by transverse aortic constriction [105]. Additionally, a novel hormone like polypeptide, irisin, can be involved in the protection of cardiac tissues during cardiac hypertension, coronary artery disease, MI, and myocardial ischemia-reperfusion injury through the inhibition of the RAAS system [106,107]. Chen and colleagues demonstrated that irisin could decrease the angiotensin II-induced cardiac fibrosis via activating the Nrf2-antioxidant signaling pathway and inhibiting pro-fibrotic TGF-β1-Smad3 signaling [107]. In a mouse model of DOX-induced cardiotoxicity, irisin ameliorated cardiac perivascular fibrosis through the inhibition of endothelial-to-mesenchymal transition by regulating ROS accumulation and autophagy disorder in endothelial cells [108]. In addition, irisin was shown to suppress ROS generation induced by angiotensin II, leading to the inhibition of its pro-fibrotic effect [107]. Furthermore, cardiac tissue fibrosis induced by angiotensin II-stimulated TGF-β enhances the binding of IL-33 to sST2 but not to ST2L, which normally provides cardioprotection against fibrotic formation. This again leads to the overproduction of angiotensin II and the subsequent progression of fibrosis formation. The inhibition of angiotensin II by angiotensin converting enzyme inhibitors reduces the inflammatory response and the level of sST2. In the absence of sST2, IL-33 interacts with ST2L [109]. Moreover, in vitro studies have shown that angiotensin II treatment significantly elevated the level of circHIPK3 in the cardiac fibroblasts and surrounding heart tissue. Thus, silencing circHIPK3 reduced the proliferation of fibroblasts and the expression of α-SMA as well as improved the diastolic functions [37]. The pathological effect of angiotensin II can be inhibited by quercetin dehydrate, which is involved in the suppression of collagen production and fibroblast proliferation and differentiation [110]. Another potential target for anti-fibrotic therapy is SNHG20, a long non-coding RNA, which is upregulated in the angiotensin II-treated murine heart. Its downregulation eliminated the angiotensin II effects on fibrosis such as the expression of fibrotic and apoptosis-related proteins by directly targeting the miR-335/Gal-3 axis [111]. In addition, the infusion of IGF-1 can improve angiotensin II-caused myocardial fibrosis via the regulation of the Akt pathway and downregulation of α-SMA expression mediated by ho-associated coiled-coil containing kinases (ROCK)2, leading to the suppression of fibroblast differentiation and proliferation [112].

Overall, despite the various previously known drugs, it seems that new RAAS inhibitors such as sacubitril/valsartan, alamandine, irisin, angiotensin converting enzyme inhibitors, and quercetin dihydrate can provide new opportunities for the treatment of cardiac fibrosis.

#### 4.4.2. TGF-β and CTGF Inhibitors

As another strategy, targeting different pro-inflammatory mediators such as TGF-β and CTGF can be utilized in order to prevent the development of cardiac fibrosis by decreasing myofibroblast activation, which results in the reduced expression of integrins by the cells, preventing further deposition of pathological ECM [29,113,114]. Recently, it was shown that the treatment of mice with CTGF monoclonal antibody, pamrevlumab, in a mouse model of MI enhanced cardiac repair and reduced adverse post-MI left ventricle remodeling [115]. The study showed a reduction in the MI-induced fibrosis in the remote, non-ischemic myocardium in mice treated with pamrevlumab compared with MI mice treated with the control IgG. Moreover, pamrevlumab reduced the basal and TGF-β1–induced α-SMA and collagen I expression, possibly through regulating the JNK signaling pathway and genes related to fibrosis and/or inflammation and cardiac repair. Another drug called pirfenidone, which was previously approved for the treatment of idiopathic pulmonary fibrosis, demonstrated promising results in the treatment of cardiac fibrosis in animal models due to its similarity to the mechanisms of myocardial and pulmonary fibrosis [116]. Several groups have reported that pirfenidone blunts TGF-β signaling, which is a crucial determinant of pulmonary and cardiac fibrosis [117]. The expansion of cardiac fibrosis following MI is thought to affect viable tissue adjacent to the infarcted area, predisposing to impulse fragmentation and the development of ventricular arrhythmias. In a rat model, pirfenidone treatment was initiated one week after ischemia-reperfusion injury and continued for four weeks. Pirfenidone caused a reduction in the scar size and myocardial fibrosis in the border zone, with better preserved left ventricle systolic function and lower rates of ventricular tachycardia inducibility [118].

In rats subjected to ligation of the left anterior descending coronary artery, pirfenidone reduced cardiac fibrosis and scar size and slowed down progression toward heart failure [119]. Moreover, pirfenidone attenuated left ventricle remodeling and improved survival in mice with diphtheria toxin-mediated acute myocardial injury and closed-chest ischemia-reperfusion injury [120]. The authors observed a reduced percentage of B lymphocytes in mice treated with pirfenidone and the depletion of B lymphocytes abolished the beneficial effects of pirfenidone. Moreover, activation of B lymphocytes that were stimulated with lipopolysaccharide and extracts of necrotic cells were attenuated by pirfenidone through a TIRAP-dependent pathway. The authors then postulated that the cardioprotective effects of pirfenidone depended at least partially on the modulation of myocardial B lymphocytes [120].

A recent study revealed that the activation of TGF-β in cardiac fibroblasts, which resulted in their transdifferentiation into myofibroblasts, is induced by the overexpression of ADAMTS16, an extracellular enzyme that is associated with ECM protein degradation and remodeling. ADAMTS16 activates the latency-associated peptide (LAP)-(TGF)-β (LAP-TGF-β) signaling pathway through the RRFR motif [121]. Another study focused on the relationship between the TGF-β/Smad2/3 signaling pathways and the Smad7 molecule. They revealed that Smad7 had an inhibitory effect on TGF-β expression in fibroblasts by promoting TβR and R-Smad turnover, leading to the downregulation of Smad2 and Smad3 molecules. This resulted in reduced myofibroblastic activity and decreased synthesis of EMPs [122]. Additionally, the TGF-β1/Smad signaling pathways are regulated by Slit2. The Slit2–Robo1 signaling pathway interferes with the functions of TGF-β1/Smad, leading to the increased production of Periostin, Robo1, and collagen I. Thus, silencing the Slit2–Robo1 pathway may open a window to a new therapy for the treatment of cardiac fibrosis [45].

The TGF-β1/Smads signaling pathway can be suppressed by tissue nonspecific alkaline phosphatase (TNAP). TNAP is overproduced in patients with MI, and the suppression of its production reduces the expression of collagen-related genes. A study on rats with MI-induced fibrosis showed that this effect was partially caused by the deactivation of the TGF-β1/Smads pathway [123]. In addition, baicalin, a natural polyphenol that has been used in traditional Chinese medicine to treat inflammation and hypertension, has recently received attention for its protective effects on CVDs such as its inhibition of the progression of atherosclerosis, against myocardial ischemia-reperfusion injury and the suppression of endothelial dysfunction. Xiao and colleagues revealed that baicalin inhibits cell proliferation, collagen synthesis, fibronectin, and CTGF protein expression in cardiac fibroblasts induced by angiotensin II in the model of abdominal aortic constriction ameliorating cardiac fibrosis in rats [124]. Moreover, baicalin inhibited the TGF-β/Smads signaling pathway stimulated with Ang II through the activation of AMPK, providing evidence that its effect against cardiac fibrosis may be attributed to its regulation in AMPK/TGF-β/Smads signaling. Finally, a recent in vivo and ex vivo study found that CTRP9 (C1q/tumor necrosis factor-related protein-9), a secreted glycoprotein highly expressed in the heart, can also be effective in reducing arterial inflammation and fibrosis, possibly via its inhibitory effects on TGF-β and collagen deposition, in the early phase of MI. It attenuated left atrial fibrosis by reducing the expressions of collagen types I and III, α-SMA, and TGF-β1 seven days after MI, possibly through depressing the Toll-like receptor 4/nuclear factor-κB and Smad2/3 signaling pathways [125].

Taken together, the inhibition of CTGF and TGF-β with their inhibitors such as CTGF mAb, pirfenidone, TNAP inhibitor, baicalin, and CTRP9 can be a promising strategy for the treatment of cardiac fibrosis due to their effects on different signaling pathways involved in the development of fibrosis in cardiac tissue.

#### 4.4.3. Gal-3 and NLRP3 Inflammasome Inhibitors

Gal-3 is a soluble β-galactosidase-binding glycoprotein. Activated macrophages and pathologically damaged cardiomyocytes are the source of their high expression in blood serum in various cardiac and cerebrovascular diseases including acute ischemic stroke, atrial fibrillation, myocardial fibrosis, and HF [126]. A recent study on rabbits revealed that treatment with Gal-3 inhibitor, modified citrus pectin (MCP), can decrease myocardial fibrosis, resulting in relatively regular and neatly arranged non-fibrotic myocardial cells with scattered nuclei in the infarct zone. In addition, myocardial Gal-3, collagen type I, the collagen type III gene, and the protein expression levels were also decreased in the MCP treatment group [127]. Moreover, in a mouse model of isoproterenol induced HF, MCP ameliorated myocardial fibrosis via the inhibition of the TLR4/MyD88/NF-κB signaling pathway and decreased expression of IL-1β, IL-18, and TNF-α, which is involved in the pathogenesis of HF [128]. On the other hand, the formation of inflammasome in the mouse MI heart can lead to an additional loss of functional myocardium, resulting in the development of HF [129]. Particularly, the NLRP3 inflammasome plays a pivotal role in the identification of danger signals and the further induction of sterile inflammatory responses after MI. Furthermore, MI-induced myocardial injury initiates the assembly of NLRP3 inflammasome, leading to the secretion of various inflammatory factors including IL-1β and IL-18. This aggravates myocardial damage and the further development of systolic dysfunction [130]. Therefore, NLRP3 inflammasome inhibitors can serve as another approach to control the development of cardiac remodeling and fibrosis. A recent study revealed that MCC950, a specific inhibitor of the NLRP3 inflammasome, can attenuate myocardial fibrosis and improve cardiac remodeling in a mouse model of MI [131]. In this study, myocardial fibrosis was reduced in the MCC950-treated animals (MCC950, 23.2 ± 3.0 vs. PBS, 36.2 ± 3.7; *p* < 0.05). Moreover, histological and molecular analysis revealed a decrease in the level of inflammatory cells in the treated group, and cardiac function was preserved compared to that in the control group. In vitro, MCC950 also inhibited NLRP3 and reduced caspase-1 activity with further downregulation of IL-1β and IL-18. Another NLRP3 inhibitor, oridonin, which is an active ingredient of medicinal herb *rabdosia rubescens*, also has an anti-inflammatory effect and suppresses NLPR3 activation by forming a covalent bond with NACHT’s cysteine 279 and preventing NEK7–NLRP3 interaction [132]. Recently, Gao and colleagues demonstrated that oridonin treatment can preserve ejection fraction and fractional shortening of the left ventricle, and notably reduce myocardial fibrosis in the treated mice. Furthermore, oridonin decreased the expression of IL-1β and IL-18 as well as myocardial macrophage and neutrophil influxes [133]. Overall, the application of Gal-3 and NLRP3 inhibitors in the treatment of cardiac fibrosis can be a promising alternative therapy in addition to the methods discussed above due to their negative effect on pro-inflammatory cytokines and pro-fibrotic genes and proteins.

#### 4.4.4. β-Adrenergic Receptor Inhibitors

β-adrenergic receptors (βARs) are members of the G-protein-coupled receptor family and consist of three subtypes: β1AR, β2AR, and β3AR. It is probable that the β3AR isoform expression in cardiac myocytes influences the cardiovascular physiology and pathology due to its lack of G protein–coupled receptor kinase recognition sites, which protect the receptor from desensitization. Receptor desensitization, in turn, is one of the pathogenetic mechanisms of chronic cardiac disease progression. Therefore, the β3AR isoform, which is resistant to desensitization, appears as a promising target for the therapy of various cardiac conditions [134,135]. Particularly, β3AR may play different roles in the regulation of cardiac contractility, relaxation, vasodilatation, and metabolism [134]. Moreover, β3AR can demonstrate protective effects against myocardial interstitial fibrosis in response to hemodynamic stress through modulating nitric oxide and oxidant stress-dependent paracrine signaling to the fibroblasts [135]. In the mouse model of MI, Niu and colleagues showed that stimulation of β3AR with its agonist BRL37344 (BRL) significantly attenuated fibrosis and decreased the scar area [136]. Further analysis revealed that BRL treatment altered the phosphorylation of endothelial nitric oxide synthase (NOS) and increased neuronal NOS expression, suggesting that the activation of both endothelial and neuronal NOS can be associated with cardioprotective effects of the receptor. However, a recent study with mirabegron, the first-in-class β3AR agonist approved for the treatment of overactive bladder in humans, demonstrated that it does not reduce infarct size and LV fraction in the swine model of reperfused MI [137]. Overall, the stimulation of β3AR can be another new approach for the treatment of myocardial fibrosis, but its agonists should be further investigated.

Thus, CTGF, TGF-β, RAAS, Gal-3, and βARs have been identified as potential mediators of fibrotic cardiac remodeling. In addition, the role of NLRP3 inflammasome in the progression of myocardial fibrosis has also been demonstrated in in vitro and in vivo studies. Hence, it is reasonable to suggest that targeting these pro-fibrotic factors with CTGF, TGF-β, RAAS, Gal-3, NLRP3, and βAR inhibitors can be a promising approach for the treatment of myocardial fibrosis after cardiac tissue damage/remodeling. Table 3 summarizes the various medications for the therapy of cardiac fibrosis.

## 5. Conclusions

Fibrosis is a pathogenetic process that leads to heart failure and increased morbidity and mortality after MI and other CVD. Unfortunately, there are no approved therapies that would specifically target cardiac fibrosis. Nevertheless, a variety of novel treatments have demonstrated encouraging results in pre-clinical studies. For instance, miRNAs and epigenetic regulators such as HDAC and methyltransferases have been shown to control fibrotic cardiac remodeling and have therefore been tested as potential candidates or targets for anti-fibrosis therapy for a number of heart conditions. CRISPR technology has been utilized to silence fibrosis-related genes as well as to genetically engineer stem cells to enhance their efficiency. Anti-fibrotic medications such as pirfenidone, angiotensin receptor blockers, NLRP3 inflammasome inhibitors, and others have either been approved or are being evaluated in clinical trials for the treatment of fibrosis-related diseases. Although their use to address cardiac fibrosis is still limited, a number of pre-clinical studies have demonstrated compelling evidence of their efficiency.

Despite promising results in pre-clinical studies, there are multiple challenges that can compromise the clinical translation of the aforementioned novel treatments. Some of these challenges, namely, low concentration at the target site, feasible route of delivery, appropriate time of administration, and the limitations of existing animal models also pertain to other known cardiac therapies. Additional hurdles concern specific therapies such as potential off-target effects in the case of CRISPR and miRNA. Possible solutions to these issues include the synergistic use of several therapeutic approaches together, the establishment of more rigorous administration protocols, the utilization of human organoids for CVD models, in silico testing, and bioinformatics tools to prevent off-site mutagenesis and many others.

## Figures and Tables

**Table 2:** The pro- and anti-fibrotic microRNAs used for the treatment of cardiac fibrosis.

Class of Micro-RNA	Example	Treatment	Outcome	Reference
Pro-fibrotic	MiR-27b	Inhibition	Lowered expression of collagen I and III, and MMP9; significantly reduced fibrotic tissue formation in rat MI model	[86]
	MiR-96	Inhibition	Suppressed angiotensin II-induced proliferation of, migration of and collagen production by murine cardiac fibroblasts; decreased inflammatory cells infiltration; and reduced collagen I and III deposition in atrial tissue	[87]
	MiR-1202	Inhibition	Decreased expression of collagen I and III, α-SMA, and Smad2/3	[89]
	MiR-150	Inhibition	Reduced expression of col1α1, col1α2, col3, and α-SMA; improved survival of cardiomyocytes in rat model of MI	[92]
Anti-fibrotic	MiR-150-5p	Use of miRNA mimic	Suppressed the expression of MMP-13, collagen I and III; induced apoptosis of human myocardial fibrosis cells after Trypanosoma cruzi infection	[93]
	MiR-1954	Overexpression	Alleviated fibrotic cardiac remodeling, reduced systolic blood pressure, and decreased the expression of cardiac hypertrophy (NppA, NppB, beta-MHC) and fibrotic marker genes (col1a1, col3a1 and col4) in angiotensin II-induced model of cardiac hypertrophy in mice	[94]
	MiR-489	Use of miRNA mimic	Downregulated pro-fibrotic markers such as col1a1, α-SMA, and HDAC2 in an isoproterenol-induced rat model of cardiac fibrosis and inhibited the viability and differentiation of cardiac fibroblasts in vitro	[95]
	MiR-30d	Overexpression	Reduced cardiac fibrosis, downregulated fibrosis marker genes such as α-SMA, inhibited cardiomyocyte apoptosis, and improved left ventricular function in a murine model of MI	[96]
	MiR-145	Overexpression	Reduced fibrous tissue formation and collagen synthesis; enhanced left ventricular function in rat model of MI	[97]

**Table 3:** The anti-fibrotic medications.

Group of Inhibitors	Drugs/Inhibitors	Outcome	Reference
RAAS inhibitors	Sacubitril/Valsartan	Blocked cardiac fibroblasts activation and proliferation via downregulation of exosomal miR-181a	[101,102,103]
	Alamandine	Decreased the density of cardiac fibrosis and reduced expression of fibrotic proteins, increased of ERK1/2 phosphorylation and restored the level of AMPKα phosphorylation preventing cardiac hypertrophy and fibrosis	[104,105]
	Irisin	Ameliorated cardiac perivascular fibrosis via regulating ROS accumulation and activating Nrf2-antioxidant signaling pathway and inhibiting pro-fibrotic TGFβ1-Smad3 signaling	[107,108]
	Angiotensin converting enzyme inhibitors	Reduced the proliferation of fibroblasts and expression of a-SMA as well as improved diastolic functions, reduced the inflammatory response and the level of sST2 providing cardioprotection against fibrosis	[37,109]
TGF-beta and CTGF inhibitors	Pamrevlumab	Reduced MI-induced fibrosis in in the remote, nonischemic myocardium, reduced basal and TGF-β1–induced αSMA and collagen-1 expression, and genes related to fibrosis	[115]
	Pirfenidone	Reduced scar size and myocardial fibrosis in the border zone, with better preserved LV systolic function and slowed down the progression toward HF	[118,119]
	Tetramisole	Reduced the expression of collagen-related genes and MI-induced fibrosis	[123]
	Baicalin	Inhibited cell proliferation, collagen synthesis, fibronectin, and CTGF protein expression in cardiac fibroblasts through reduction of TGF-β/Smads signaling pathway	[124]
	C1q/tumor necrosis factor-related protein-9	Reduced atrial inflammation and fibrosis via inhibition of TLR4/MyD88/NF-κB signaling pathway, TGF-β, collagen deposition, in early phase of MI, decreased the expression of IL-1β, IL-18 and TNF-α	[127,128]
Galectin-3 and NLRP3 inflammasome Inhibitor	Modified citrus pectin	Decreased myocardial fibrosis, myocardial Gal-3, collagen type I, and collagen type III gene and protein expressions via inhibiting TLR4/MyD88/NF-κB signaling pathway, decreased expression of IL-1β, IL-18, and TNF-α	[127,128]
	MCC950	Attenuated myocardial fibrosis and improved cardiac remodeling as well as inhibited NLRP3 and reduced caspase-1 activity with further downregulation of IL-1β and IL-18	[131]
	Oridonin	Reduced myocardial fibrosis, decreased expression of IL-1β and IL-18 as well as infiltration by myocardial macrophages and neutrophils	[133]
β3AR inhibitors	BRL37344 Agonist	Attenuated fibrosis, decreased scar area, altered the phosphorylation of endothelial NOS, and increased neuronal NOS expression	[136]

## References

[1] Virani S.S., Alonso A., Benjamin E.J., Bittencourt M.S., Callaway C.W., Carson A.P., Chamberlain A.M., Chang A.R., Cheng S., Delling F.N. (2020). Heart disease and stroke statistics—2020 update: A report from the American Heart Association. Circulation.

[2] Timmis A., Vardas P., Townsend N., Torbica A., Katus H., De Smedt D., Gale C.P., Maggioni A.P., Petersen S.E., Huculeci R. (2022). European Society of Cardiology: Cardiovascular disease statistics 2021. Eur. Heart J..

[3] Weiskirchen R., Weiskirchen S., Tacke F. (2019). Organ and tissue fibrosis: Molecular signals, cellular mechanisms and translational implications. Mol. Asp. Med..

[4] Klinkhammer B.M., Floege J., Boor P. (2018). PDGF in organ fibrosis. Mol. Asp. Med..

[5] Hashimoto H., Olson E.N., Bassel-Duby R. (2018). Therapeutic approaches for cardiac regeneration and repair. Nat. Rev. Cardiol..

[6] Raziyeva K., Smagulova A., Kim Y., Smagul S., Nurkesh A., Saparov A. (2020). Preconditioned and genetically modified stem cells for myocardial infarction treatment. Int. J. Mol. Sci..

[7] Kim Y., Zharkinbekov Z., Sarsenova M., Yeltay G., Saparov A. (2021). Recent Advances in Gene Therapy for Cardiac Tissue Regeneration. Int. J. Mol. Sci..

[8] Hinderer S., Schenke-Layland K. (2019). Cardiac fibrosis—A short review of causes and therapeutic strategies. Adv. Drug Deliv. Rev..

[9] De Boer R.A., Heymans S., Backs J., Carrier L., Coats A.J., Dimmeler S., Eschenhagen T., Filippatos G., Gepstein L., Hulot J.S. (2022). Targeted therapies in genetic dilated and hypertrophic cardiomyopathies: From molecular mechanisms to therapeutic targets. A position paper from the Heart Failure Association (HFA) and the Working Group on Myocardial Function of the European Society of Cardiology (ESC). Eur. J. Heart Fail..

[10] Raafs A.G., Verdonschot J.A., Henkens M.T., Adriaans B.P., Wang P., Derks K., Abdul Hamid M.A., Knackstedt C., van Empel V.P., Díez J. (2021). The combination of carboxy-terminal propeptide of procollagen type I blood levels and late gadolinium enhancement at cardiac magnetic resonance provides additional prognostic information in idiopathic dilated cardiomyopathy–A multilevel assessment of myocardial fibrosis in dilated cardiomyopathy. Eur. J. Heart Fail..

[11] Zhao X., Kwan J.Y.Y., Yip K., Liu P.P., Liu F.-F. (2020). Targeting metabolic dysregulation for fibrosis therapy. Nat. Rev. Drug Discov..

[12] Park S., Nguyen N.B., Pezhouman A., Ardehali R. (2019). Cardiac fibrosis: Potential therapeutic targets. Transl. Res..

[13] Ma Z.G., Yuan Y.P., Wu H.M., Zhang X., Tang Q.Z. (2018). Cardiac fibrosis: New insights into the pathogenesis. Int. J. Biol. Sci..

[14] Kim Y., Nurakhayev S., Nurkesh A., Zharkinbekov Z., Saparov A. (2021). Macrophage Polarization in Cardiac Tissue Repair following Myocardial Infarction. Int. J. Mol. Sci..

[15] Gibb A.A., Lazaropoulos M.P., Elrod J.W. (2020). Myofibroblasts and fibrosis: Mitochondrial and metabolic control of cellular differentiation. Circ. Res..

[16] Disertori M., Masè M., Ravelli F. (2017). Myocardial fibrosis predicts ventricular tachyarrhythmias. Trends Cardiovasc. Med..

[17] Hall C., Gehmlich K., Denning C., Pavlovic D. (2021). Complex relationship between cardiac fibroblasts and cardiomyocytes in health and disease. J. Am. Heart Assoc..

[18] Park T.-J., Park J.H., Lee G.S., Lee J.-Y., Shin J.H., Kim M.W., Kim Y.S., Kim J.-Y., Oh K.-J., Han B.-S. (2019). Quantitative proteomic analyses reveal that GPX4 downregulation during myocardial infarction contributes to ferroptosis in cardiomyocytes. Cell Death Dis..

[19] Giacca M. (2020). Cardiac regeneration after myocardial infarction: An approachable goal. Curr. Cardiol. Rep..

[20] Yousefi F., Shabaninejad Z., Vakili S., Derakhshan M., Movahedpour A., Dabiri H., Ghasemi Y., Mahjoubin-Tehran M., Nikoozadeh A., Savardashtaki A. (2020). TGF-β and WNT signaling pathways in cardiac fibrosis: Non-coding RNAs come into focus. Cell Commun. Signal..

[21] Bacmeister L., Schwarzl M., Warnke S., Stoffers B., Blankenberg S., Westermann D., Lindner D. (2019). Inflammation and fibrosis in murine models of heart failure. Basic Res. Cardiol..

[22] Kurose H. (2021). Cardiac fibrosis and fibroblasts. Cells.

[23] Webber M., Jackson S.P., Moon J.C., Captur G. (2020). Myocardial fibrosis in heart failure: Anti-fibrotic therapies and the role of cardiovascular magnetic resonance in drug trials. Cardiol. Ther..

[24] Karamitsos T.D., Arvanitaki A., Karvounis H., Neubauer S., Ferreira V.M. (2020). Myocardial tissue characterization and fibrosis by imaging. Cardiovasc. Imaging.

[25] Tanaka R., Umemura M., Narikawa M., Hikichi M., Osaw K., Fujita T., Yokoyama U., Ishigami T., Tamura K., Ishikawa Y. (2020). Reactive fibrosis precedes doxorubicin-induced heart failure through sterile inflammation. ESC Heart Fail..

[26] Hara H., Takeda N., Komuro I. (2017). Pathophysiology and therapeutic potential of cardiac fibrosis. Inflamm. Regen..

[27] Kyriakou P., Mouselimis D., Tsarouchas A., Rigopoulos A., Bakogiannis C., Noutsias M., Vassilikos V. (2018). Diagnosis of cardiac amyloidosis: A systematic review on the role of imaging and biomarkers. BMC Cardiovasc. Disord..

[28] Bonderman D., Pölzl G., Ablasser K., Agis H., Aschauer S., Auer-Grumbach M., Binder C., Dörler J., Duca F., Ebner C. (2020). Diagnosis and treatment of cardiac amyloidosis: An interdisciplinary consensus statement. Wien. Klin. Wochenschr..

[29] Heymans S., González A., Pizard A., Papageorgiou A.P., López-Andrés N., Jaisser F., Thum T., Zannad F., Díez J. (2015). Searching for new mechanisms of myocardial fibrosis with diagnostic and/or therapeutic potential. Eur. J. Heart Fail..

[30] Frangogiannis N.G. (2019). Cardiac fibrosis: Cell biological mechanisms, molecular pathways and therapeutic opportunities. Mol. Asp. Med..

[31] Scharf G.M., Kilian K., Cordero J., Wang Y., Grund A., Hofmann M., Froese N., Wang X., Kispert A., Kist R. (2019). Inactivation of Sox9 in fibroblasts reduces cardiac fibrosis and inflammation. JCI Insight.

[32] Wen H., Peng L., Chen Y. (2021). The effect of immune cell-derived exosomes in the cardiac tissue repair after myocardial infarction: Molecular mechanisms and pre-clinical evidence. J. Cell. Mol. Med..

[33] Kong P., Christia P., Frangogiannis N.G. (2014). The pathogenesis of cardiac fibrosis. Cell. Mol. Life Sci..

[34] Raziyeva K., Kim Y., Zharkinbekov Z., Kassymbek K., Jimi S., Saparov A. (2021). Immunology of acute and chronic wound healing. Biomolecules.

[35] Ceauşu Z., Socea B., Costache M., Predescu D., Şerban D., Smarandache C.G., Pacu I., Alexandru H.H., Daviţoiu A.M., Jacotă-Alexe F. (2021). Fibroblast involvement in cardiac remodeling and repair under ischemic conditions. Exp. Ther. Med..

[36] Talman V., Ruskoaho H. (2016). Cardiac fibrosis in myocardial infarction—from repair and remodeling to regeneration. Cell Tissue Res..

[37] Ni H., Li W., Zhuge Y., Xu S., Wang Y., Chen Y., Shen G., Wang F. (2019). Inhibition of circHIPK3 prevents angiotensin II-induced cardiac fibrosis by sponging miR-29b-3p. Int. J. Cardiol..

[38] Krenning G., Zeisberg E.M., Kalluri R. (2010). The origin of fibroblasts and mechanism of cardiac fibrosis. J. Cell. Physiol..

[39] Frangogiannis N.G. (2021). Cardiac fibrosis. Cardiovasc. Res..

[40] Aujla P.K., Kassiri Z. (2021). Diverse origins and activation of fibroblasts in cardiac fibrosis. Cell. Signal..

[41] Dobaczewski M., de Haan J.J., Frangogiannis N.G. (2012). The extracellular matrix modulates fibroblast phenotype and function in the infarcted myocardium. J. Cardiovasc. Transl. Res..

[42] Cakir S.N., Whitehead K.M., Hendricks H.K., de Castro Brás L.E. (2022). Novel Techniques Targeting Fibroblasts after Ischemic Heart Injury. Cells.

[43] Sygitowicz G., Maciejak-Jastrzębska A., Sitkiewicz D. (2021). A review of the molecular mechanisms underlying cardiac fibrosis and atrial fibrillation. J. Clin. Med..

[44] Hamid T., Xu Y., Ismahil M.A., Rokosh G., Jinno M., Zhou G., Wang Q., Prabhu S.D. (2022). Cardiac mesenchymal stem cells promote fibrosis and remodeling in heart failure: Role of PDGF signaling. JACC Basic Transl. Sci..

[45] Liu Y., Yin Z., Xu X., Liu C., Duan X., Song Q., Tuo Y., Wang C., Yang J., Yin S. (2021). Crosstalk between the activated Slit2–Robo1 pathway and TGF-β1 signalling promotes cardiac fibrosis. ESC Heart Fail..

[46] Noskovicova N., Schuster R., van Putten S., Ezzo M., Koehler A., Boo S., Coelho N.M., Griggs D., Ruminski P., McCulloch C.A. (2021). Suppression of the fibrotic encapsulation of silicone implants by inhibiting the mechanical activation of pro-fibrotic TGF-β. Nat. Biomed. Eng..

[47] Działo E., Czepiel M., Tkacz K., Siedlar M., Kania G., Błyszczuk P. (2021). WNT/β-catenin signaling promotes TGF-β-mediated activation of human cardiac fibroblasts by enhancing IL-11 production. Int. J. Mol. Sci..

[48] Hanna A., Humeres C., Frangogiannis N.G. (2021). The role of Smad signaling cascades in cardiac fibrosis. Cell. Signal..

[49] Vallée A., Lecarpentier Y. (2019). TGF-β in fibrosis by acting as a conductor for contractile properties of myofibroblasts. Cell Biosci..

[50] Parichatikanond W., Luangmonkong T., Mangmool S., Kurose H. (2020). Therapeutic targets for the treatment of cardiac fibrosis and cancer: Focusing on TGF-β signaling. Front. Cardiovasc. Med..

[51] AlQudah M., Hale T.M., Czubryt M.P. (2020). Targeting the renin-angiotensin-aldosterone system in fibrosis. Matrix Biol..

[52] Jia G., Aroor A.R., Hill M.A., Sowers J.R. (2018). Role of renin-angiotensin-aldosterone system activation in promoting cardiovascular fibrosis and stiffness. Hypertension.

[53] Lyu X., Hu M., Peng J., Zhang X., Sanders Y.Y. (2019). HDAC inhibitors as antifibrotic drugs in cardiac and pulmonary fibrosis. Ther. Adv. Chronic Dis..

[54] Yoon S., Kang G., Eom G.H. (2019). HDAC inhibitors: Therapeutic potential in fibrosis-associated human diseases. Int. J. Mol. Sci..

[55] Fernández-Barrena M.G., Arechederra M., Colyn L., Berasain C., Avila M.A. (2020). Epigenetics in hepatocellular carcinoma development and therapy: The tip of the iceberg. JHEP Rep..

[56] Prasher D., Greenway S.C., Singh R.B. (2020). The impact of epigenetics on cardiovascular disease. Biochem. Cell Biol..

[57] Soler-Botija C., Forcales S.V., Genís A.B. (2020). Spotlight on epigenetic reprogramming in cardiac regeneration. Semin. Cell Dev. Biol..

[58] Li X., Yang Y., Chen S., Zhou J., Li J., Cheng Y. (2021). Epigenetics-based therapeutics for myocardial fibrosis. Life Sci..

[59] Wang Y., Chen P., Wang L., Zhao J., Zhong Z., Wang Y., Xu J. (2018). Inhibition of histone deacetylases prevents cardiac remodeling after myocardial infarction by restoring autophagosome processing in cardiac fibroblasts. Cell. Physiol. Biochem..

[60] Zhang L.X., Du J., Zhao Y.T., Wang J., Zhang S., Dubielecka P.M., Wei L., Zhuang S., Qin G., Chin Y.E. (2018). Transgenic overexpression of active HDAC4 in the heart attenuates cardiac function and exacerbates remodeling in infarcted myocardium. J. Appl. Physiol..

[61] Barbosa D.M., Fahlbusch P., Herzfeld de Wiza D., Jacob S., Kettel U., Al-Hasani H., Krüger M., Ouwens D.M., Hartwig S., Lehr S. (2020). Rhein, a novel Histone Deacetylase (HDAC) inhibitor with antifibrotic potency in human myocardial fibrosis. Sci. Rep..

[62] Li F., Li L., Zhang J., Yang X., Liu Y. (2022). Histone methyltransferase DOT1L mediates the TGF-β1/Smad3 signaling pathway through epigenetic modification of SYK in myocardial infarction. Hum. Cell.

[63] Sung P.-H., Luo C.-W., Chiang J.Y., Yip H.-K. (2020). The combination of G9a histone methyltransferase inhibitors with erythropoietin protects heart against damage from acute myocardial infarction. Am. J. Transl. Res..

[64] Liu F., Wen Y., Kang J., Wei C., Wang M., Zheng Z., Peng J. (2018). Regulation of TLR4 expression mediates the attenuating effect of erythropoietin on inflammation and myocardial fibrosis in rat heart. Int. J. Mol. Med..

[65] Yu L., Wang L., Yi H., Wu X. (2020). LRP6-CRISPR prevents activation of hepatic stellate cells and liver fibrogenesis in rats. Am. J. Transl. Res..

[66] Yu L., Wang L., Wu X., Yi H. (2021). RSPO4-CRISPR alleviates liver injury and restores gut microbiota in a rat model of liver fibrosis. Commun. Biol..

[67] Xu X., Tan X., Tampe B., Wilhelmi T., Hulshoff M.S., Saito S., Moser T., Kalluri R., Hasenfuss G., Zeisberg E.M. (2018). High-fidelity CRISPR/Cas9-based gene-specific hydroxymethylation rescues gene expression and attenuates renal fibrosis. Nat. Commun..

[68] Tan Q., Link P.A., Meridew J.A., Pham T.X., Caporarello N., Ligresti G., Tschumperlin D.J. (2021). Spontaneous lung fibrosis resolution reveals novel antifibrotic regulators. Am. J. Respir. Cell Mol. Biol..

[69] Nishiga M., Liu C., Qi L.S., Wu J.C. (2022). The use of new CRISPR tools in cardiovascular research and medicine. Nat. Rev. Cardiol..

[70] Park H., Kim D., Cho B., Byun J., Kim Y.S., Ahn Y., Hur J., Oh Y.-K., Kim J. (2022). In vivo therapeutic genome editing via CRISPR/Cas9 magnetoplexes for myocardial infarction. Biomaterials.

[71] Jiang L., Liang J., Huang W., Ma J., Park K.H., Wu Z., Chen P., Zhu H., Ma J.-J., Cai W. (2022). CRISPR activation of endogenous genes reprograms fibroblasts into cardiovascular progenitor cells for myocardial infarction therapy. Mol. Ther..

[72] Cho H.-M., Lee K.-H., Shen Y.-M., Shin T.-J., Ryu P.-D., Choi M.-C., Kang K.-S., Cho J.-Y. (2020). Transplantation of hMSCs genome edited with LEF1 improves cardio-protective effects in myocardial infarction. Mol. Ther. Nucleic Acids.

[73] Meng X., Zheng M., Yu M., Bai W., Zuo L., Bu X., Liu Y., Xia L., Hu J., Liu L. (2019). Transplantation of CRISPRa system engineered IL10-overexpressing bone marrow-derived mesenchymal stem cells for the treatment of myocardial infarction in diabetic mice. J. Biol. Eng..

[74] Liu Y., Xu J., Wu M., Kang L., Xu B. (2020). The effector cells and cellular mediators of immune system involved in cardiac inflammation and fibrosis after myocardial infarction. J. Cell. Physiol..

[75] Rezaei H., Farahani N., Hosseingholi E.Z., Sathyapalan T., hossein Sahebkar A. (2020). Harnessing CRISPR/Cas9 technology in cardiovascular disease. Trends Cardiovasc. Med..

[76] Dexheimer P.J., Cochella L. (2020). MicroRNAs: From Mechanism to Organism. Front. Cell Dev. Biol..

[77] Ferrari S., Pesce M. (2019). Cell-Based Mechanosensation, Epigenetics, and Non-Coding RNAs in Progression of Cardiac Fibrosis. Int. J. Mol. Sci..

[78] Varzideh F., Kansakar U., Donkor K., Wilson S., Jankauskas S.S., Mone P., Wang X., Lombardi A., Santulli G. (2022). Cardiac Remodeling After Myocardial Infarction: Functional Contribution of microRNAs to Inflammation and Fibrosis. Front. Cardiovasc. Med..

[79] O’Reilly S. (2016). MicroRNAs in fibrosis: Opportunities and challenges. Arthritis Res. Ther..

[80] Ghafouri-Fard S., Abak A., Talebi S.F., Shoorei H., Branicki W., Taheri M., Dilmaghani N.A. (2021). Role of miRNA and lncRNAs in organ fibrosis and aging. Biomed. Pharmacother..

[81] Liu X., Xu Y., Deng Y., Li H. (2018). MicroRNA-223 regulates cardiac fibrosis after myocardial infarction by targeting RASA1. Cell. Physiol. Biochem..

[82] Liu Y., Song J.-W., Lin J.-Y., Miao R., Zhong J.-C. (2020). Roles of microRNA-122 in cardiovascular fibrosis and related diseases. Cardiovasc. Toxicol..

[83] Peters L.J.F., Biessen E.A.L., Hohl M., Weber C., van der Vorst E.P.C., Santovito D. (2020). Small Things Matter: Relevance of MicroRNAs in Cardiovascular Disease. Front. Physiol..

[84] Li G., Shao Y., Guo H.C., Zhi Y., Qiao B., Ma K., Lai Y.Q., Du J., Li Y. (2021). MicroRNA-27b-3p downregulates FGF1 and aggravates pathological cardiac remodelling. Cardiovasc. Res..

[85] Chatterjee E., Das S. (2022). Non-coding RNAs in cardiac remodeling: Diversity in composition and function. Curr. Opin. Physiol..

[86] Fu Q., Lu Z., Fu X., Ma S., Lu X. (2019). MicroRNA 27b promotes cardiac fibrosis by targeting the FBW7/Snail pathway. Aging.

[87] Su L., Yao Y., Song W. (2020). Downregulation of miR-96 suppresses the profibrogenic functions of cardiac fibroblasts induced by angiotensin II and attenuates atrial fibrosis by upregulating KLF13. Hum. Cell.

[88] Yu Y.H., Zhang Y.H., Ding Y.Q., Bi X.Y., Yuan J., Zhou H., Wang P.X., Zhang L.L., Ye J.T. (2021). MicroRNA-99b-3p promotes angiotensin II-induced cardiac fibrosis in mice by targeting GSK-3β. Acta Pharmacol. Sin..

[89] Xiao J., Zhang Y., Tang Y., Dai H., OuYang Y., Li C., Yu M. (2021). MiRNA-1202 promotes the TGF-β1-induced proliferation, differentiation and collagen production of cardiac fibroblasts by targeting nNOS. PLoS ONE.

[90] Chen C., Ponnusamy M., Liu C., Gao J., Wang K., Li P. (2017). MicroRNA as a Therapeutic Target in Cardiac Remodeling. BioMed Res. Int..

[91] Yuan J., Liu H., Gao W., Zhang L., Ye Y., Yuan L., Ding Z., Wu J., Kang L., Zhang X. (2018). MicroRNA-378 suppresses myocardial fibrosis through a paracrine mechanism at the early stage of cardiac hypertrophy following mechanical stress. Theranostics.

[92] Tian H.B., Li S.H., Hu K.Q., Zan Y.S., Zhang X.L., Su G.H. (2019). MicroRNA-150 alleviates acute myocardial infarction through regulating cardiac fibroblasts in ventricular remodeling. Eur. Rev. Med. Pharmacol. Sci..

[93] Shen J., Xing W., Gong F., Wang W., Yan Y., Zhang Y., Xie C., Fu S. (2019). MiR-150-5p retards the progression of myocardial fibrosis by targeting EGR1. Cell Cycle.

[94] Chiasson V., Takano A.P.C., Guleria R.S., Gupta S. (2019). Deficiency of MicroRNA miR-1954 Promotes Cardiac Remodeling and Fibrosis. J. Am. Heart Assoc..

[95] Yang X., Yu T., Zhang S. (2020). MicroRNA-489 suppresses isoproterenol-induced cardiac fibrosis by downregulating histone deacetylase 2. Exp. Ther. Med..

[96] Li J., Salvador A.M., Li G., Valkov N., Ziegler O., Yeri A., Yang Xiao C., Meechoovet B., Alsop E., Rodosthenous R.S. (2021). Mir-30d Regulates Cardiac Remodeling by Intracellular and Paracrine Signaling. Circ. Res..

[97] Cui S., Liu Z., Tao B., Fan S., Pu Y., Meng X., Li D., Xia H., Xu L. (2021). miR-145 attenuates cardiac fibrosis through the AKT/GSK-3β/β-catenin signaling pathway by directly targeting SOX9 in fibroblasts. J. Cell. Biochem..

[98] Liu M., de Juan Abad B.L., Cheng K. (2021). Cardiac fibrosis: Myofibroblast-mediated pathological regulation and drug delivery strategies. Adv. Drug Deliv. Rev..

[99] Leader C.J., Moharram M., Coffey S., Sammut I.A., Wilkins G.W., Walker R.J. (2019). Myocardial global longitudinal strain: An early indicator of cardiac interstitial fibrosis modified by spironolactone, in a unique hypertensive rat model. PLoS ONE.

[100] Kovács Z.Z., Szűcs G., Freiwan M., Kovács M.G., Márványkövi F.M., Dinh H., Siska A., Farkas K., Kovács F., Kriston A. (2021). Comparison of the antiremodeling effects of losartan and mirabegron in a rat model of uremic cardiomyopathy. Sci. Rep..

[101] Khder Y., Shi V., McMurray J.J.V., Lefkowitz M.P. (2017). Sacubitril/Valsartan (LCZ696) in Heart Failure. Handb. Exp. Pharmacol..

[102] Burke R.M., Lighthouse J.K., Mickelsen D.M., Small E.M. (2019). Sacubitril/Valsartan Decreases Cardiac Fibrosis in Left Ventricle Pressure Overload by Restoring PKG Signaling in Cardiac Fibroblasts. Circ. Heart Fail..

[103] Vaskova E., Ikeda G., Tada Y., Wahlquist C., Mercola M., Yang P.C. (2020). Sacubitril/Valsartan Improves Cardiac Function and Decreases Myocardial Fibrosis Via Downregulation of Exosomal miR-181a in a Rodent Chronic Myocardial Infarction Model. J. Am. Heart Assoc..

[104] Wang L., Liu C., Chen X., Li P. (2019). Alamandine attenuates long-term hypertension-induced cardiac fibrosis independent of blood pressure. Mol. Med. Rep..

[105] Silva M.M., de Souza-Neto F.P., Jesus I.C.G., Gonçalves G.K., Santuchi M.C., Sanches B.L., de Alcântara-Leonídio T.C., Melo M.B., Vieira M.A.R., Guatimosim S. (2021). Alamandine improves cardiac remodeling induced by transverse aortic constriction in mice. Am. J. Physiol. Heart Circ. Physiol..

[106] Fu J., Li F., Tang Y., Cai L., Zeng C., Yang Y., Yang J. (2021). The Emerging Role of Irisin in Cardiovascular Diseases. J. Am. Heart Assoc..

[107] Chen R.R., Fan X.H., Chen G., Zeng G.W., Xue Y.G., Liu X.T., Wang C.Y. (2019). Irisin attenuates angiotensin II-induced cardiac fibrosis via Nrf2 mediated inhibition of ROS/ TGFβ1/Smad2/3 signaling axis. Chem. Biol. Interact..

[108] Pan J.A., Zhang H., Lin H., Gao L., Zhang H.L., Zhang J.F., Wang C.Q., Gu J. (2021). Irisin ameliorates doxorubicin-induced cardiac perivascular fibrosis through inhibiting endothelial-to-mesenchymal transition by regulating ROS accumulation and autophagy disorder in endothelial cells. Redox Biol..

[109] Ambari A.M., Setianto B., Santoso A., Radi B., Dwiputra B., Susilowati E., Tulrahmi F., Doevendans P.A., Cramer M.J. (2020). Angiotensin Converting Enzyme Inhibitors (ACEIs) Decrease the Progression of Cardiac Fibrosis in Rheumatic Heart Disease Through the Inhibition of IL-33/sST2. Front. Cardiovasc. Med..

[110] Wang L., Tan A., An X., Xia Y., Xie Y. (2020). Quercetin Dihydrate inhibition of cardiac fibrosis induced by angiotensin II In Vivo and In Vitro. Biomed. Pharm..

[111] Li M., Qi C., Song R., Xiong C., Zhong X., Song Z., Ning Z., Song X. (2021). Inhibition of Long Noncoding RNA SNHG20 Improves Angiotensin II-Induced Cardiac Fibrosis and Hypertrophy by Regulating the MicroRNA 335/Galectin-3 Axis. Mol. Cell Biol..

[112] Ock S., Ham W., Kang C.W., Kang H., Lee W.S., Kim J. (2021). IGF-1 protects against angiotensin II-induced cardiac fibrosis by targeting αSMA. Cell Death Dis..

[113] Lipson K.E., Wong C., Teng Y., Spong S. (2012). CTGF is a central mediator of tissue remodeling and fibrosis and its inhibition can reverse the process of fibrosis. Fibrogenesis Tissue Repair.

[114] Meagher P.B., Lee X.A., Lee J., Visram A., Friedberg M.K., Connelly K.A. (2021). Cardiac fibrosis: Key role of integrins in cardiac homeostasis and remodeling. Cells.

[115] Vainio L.E., Szabó Z., Lin R., Ulvila J., Yrjölä R., Alakoski T., Piuhola J., Koch W.J., Ruskoaho H., Fouse S.D. (2019). Connective Tissue Growth Factor Inhibition Enhances Cardiac Repair and Limits Fibrosis After Myocardial Infarction. JACC Basic Transl. Sci..

[116] Aimo A., Cerbai E., Bartolucci G., Adamo L., Barison A., Lo Surdo G., Biagini S., Passino C., Emdin M. (2020). Pirfenidone is a cardioprotective drug: Mechanisms of action and preclinical evidence. Pharmacol. Res..

[117] Graziani F., Varone F., Crea F., Richeldi L. (2018). Treating heart failure with preserved ejection fraction: Learning from pulmonary fibrosis. Eur. J. Heart Fail..

[118] Nguyen D.T., Ding C., Wilson E., Marcus G.M., Olgin J.E. (2010). Pirfenidone mitigates left ventricular fibrosis and dysfunction after myocardial infarction and reduces arrhythmias. Heart Rhythm..

[119] Li C., Han R., Kang L., Wang J., Gao Y., Li Y., He J., Tian J. (2017). Pirfenidone controls the feedback loop of the AT1R/p38 MAPK/renin-angiotensin system axis by regulating liver X receptor-α in myocardial infarction-induced cardiac fibrosis. Sci. Rep..

[120] Adamo L., Staloch L.J., Rocha-Resende C., Matkovich S.J., Jiang W., Bajpai G., Weinheimer C.J., Kovacs A., Schilling J.D., Barger P.M. (2018). Modulation of subsets of cardiac B lymphocytes improves cardiac function after acute injury. JCI Insight.

[121] Yao Y., Hu C., Song Q., Li Y., Da X., Yu Y., Li H., Clark I.M., Chen Q., Wang Q.K. (2020). ADAMTS16 activates latent TGF-β, accentuating fibrosis and dysfunction of the pressure-overloaded heart. Cardiovasc. Res..

[122] Humeres C., Shinde A.V., Hanna A., Alex L., Hernández S.C., Li R., Chen B., Conway S.J., Frangogiannis N.G. (2022). Smad7 effects on TGF-β and ErbB2 restrain myofibroblast activation and protect from postinfarction heart failure. J. Clin. Investig..

[123] Gao L., Wang L.Y., Liu Z.Q., Jiang D., Wu S.Y., Guo Y.Q., Tao H.M., Sun M., You L.N., Qin S. (2020). TNAP inhibition attenuates cardiac fibrosis induced by myocardial infarction through deactivating TGF-β1/Smads and activating P53 signaling pathways. Cell Death Dis..

[124] Xiao Y., Ye J., Zhou Y., Huang J., Liu X., Huang B., Zhu L., Wu B., Zhang G., Cai Y. (2018). Baicalin inhibits pressure overload-induced cardiac fibrosis through regulating AMPK/TGF-β/Smads signaling pathway. Arch. Biochem. Biophys..

[125] Liu M., Li W., Wang H., Yin L., Ye B., Tang Y., Huang C. (2019). CTRP9 Ameliorates Atrial Inflammation, Fibrosis, and Vulnerability to Atrial Fibrillation in Post-Myocardial Infarction Rats. J. Am. Heart Assoc..

[126] Cao Z.Q., Yu X., Leng P. (2021). Research progress on the role of gal-3 in cardio/cerebrovascular diseases. Biomed. Pharm..

[127] Li S., Li S., Hao X., Zhang Y., Deng W. (2019). Perindopril and a Galectin-3 Inhibitor Improve Ischemic Heart Failure in Rabbits by Reducing Gal-3 Expression and Myocardial Fibrosis. Front. Physiol..

[128] Xu G.R., Zhang C., Yang H.X., Sun J.H., Zhang Y., Yao T.T., Li Y., Ruan L., An R., Li A.Y. (2020). Modified citrus pectin ameliorates myocardial fibrosis and inflammation via suppressing galectin-3 and TLR4/MyD88/NF-κB signaling pathway. Biomed. Pharm..

[129] Mezzaroma E., Toldo S., Farkas D., Seropian I.M., Van Tassell B.W., Salloum F.N., Kannan H.R., Menna A.C., Voelkel N.F., Abbate A. (2011). The inflammasome promotes adverse cardiac remodeling following acute myocardial infarction in the mouse. Proc. Natl. Acad. Sci. USA.

[130] Zhou W., Chen C., Chen Z., Liu L., Jiang J., Wu Z., Zhao M., Chen Y. (2018). NLRP3: A Novel Mediator in Cardiovascular Disease. J. Immunol. Res..

[131] Gao R., Shi H., Chang S., Gao Y., Li X., Lv C., Yang H., Xiang H., Yang J., Xu L. (2019). The selective NLRP3-inflammasome inhibitor MCC950 reduces myocardial fibrosis and improves cardiac remodeling in a mouse model of myocardial infarction. Int. Immunopharmacol..

[132] He H., Jiang H., Chen Y., Ye J., Wang A., Wang C., Liu Q., Liang G., Deng X., Jiang W. (2018). Oridonin is a covalent NLRP3 inhibitor with strong anti-inflammasome activity. Nat. Commun..

[133] Gao R.F., Li X., Xiang H.Y., Yang H., Lv C.Y., Sun X.L., Chen H.Z., Gao Y., Yang J.S., Luo W. (2021). The covalent NLRP3-inflammasome inhibitor Oridonin relieves myocardial infarction induced myocardial fibrosis and cardiac remodeling in mice. Int. Immunopharmacol..

[134] Cannavo A., Koch W.J. (2017). Targeting β3-Adrenergic Receptors in the Heart: Selective Agonism and β-Blockade. J. Cardiovasc. Pharmacol..

[135] Michel L.Y., Farah C., Balligand J.-L. (2020). The beta3 adrenergic receptor in healthy and pathological cardiovascular tissues. Cells.

[136] Niu X., Zhao L., Li X., Xue Y., Wang B., Lv Z., Chen J., Sun D., Zheng Q. (2014). β3-Adrenoreceptor stimulation protects against myocardial infarction injury via eNOS and nNOS activation. PLoS ONE.

[137] Rossello X., Piñero A., Fernández-Jiménez R., Sánchez-González J., Pizarro G., Galán-Arriola C., Lobo-Gonzalez M., Vilchez J.P., García-Prieto J., García-Ruiz J.M. (2018). Mirabegron, a Clinically Approved β3 adrenergic receptor agonist, does not reduce infarct size in a swine model of reperfused myocardial infarction. J. Cardiovasc. Transl. Res..

